# Performance evaluation of thrombomodulin, thrombin‐antithrombin complex, plasmin‐α2‐antiplasmin complex, and t‐PA: PAI‐1 complex

**DOI:** 10.1002/jcla.22913

**Published:** 2019-05-15

**Authors:** Qian Chen, Weiling Shou, Wei Wu, Geng Wang, Wei Cui

**Affiliations:** ^1^ Department of Clinical Laboratory, Peking Union Medical College Hospital Peking Union Medical College and Chinese Academy of Medical Sciences Beijing China; ^2^ Department of Clinical Laboratory Cancer Hospital Chinese Academy of Medical Sciences Beijing China

**Keywords:** plasmin‐α2‐antiplasmin, thrombin‐antithrombin, thrombomodulin, t‐PA: PAI‐1 complex

## Abstract

**Background:**

To conduct a comprehensive performance evaluation of a fully automated analyzer for measuring thrombomodulin (TM), thrombin‐antithrombin complex (TAT), plasmin‐α2‐antiplasmin complex (PAP), and t‐PA: PAI‐1 complex (tPAI‐C).

**Methods:**

According to the Clinical and Laboratory Standards Institute (CLSI) EP05‐A2, EP06‐A specifications, TM, TAT, PAP, and tPAI‐C were analyzed to evaluate intraassay variability and interassay variability, linear range, carryover rate, reference range, sample stability, and interferences.

**Results:**

The intraassay variability and interassay variability of the four factors were all below 5%. The carryover rates were below 1%. Linear verification analysis revealed correlation coefficients of 0.998‐0.999. The recommended reference ranges of TM, TAT, and PAP were appropriate for our laboratory, whereas the reference of tPAI‐C should be established by each laboratory. Stability assessment revealed that TM is stable for 2 days at room temperature but lacks stability at colder temperatures. In contrast, TAT is stable for 5 days at 4°C and −20°C but has poor stability at room temperature. PAP and tPAI‐C are stable for 3 days at all three temperatures. The measurement of TM, TAT, PAP, and tPAI‐C is not altered by the presence of 510 mg/dL hemoglobin, 1490 FTU triglycerides, or 21.1 mg/dL conjugated and free bilirubin.

**Conclusion:**

The determination of TM, TAT, PAP, and tPAI‐C using a high‐sensitivity chemiluminescence analyzer performs well in terms of precision, carryover rate, linear range, and interference. Thus, this method is suitable for the detection of these substances in clinical specimens.

## 1 INTRODUCTION

The incidence of thrombotic diseases characterized by intravascular clotting has risen recently. The molecular markers for coagulation, fibrinolysis, and damage to the vascular endothelium have important clinical significance for detecting the generation, development, and prognosis of thrombus. However, the clinical application of certain early‐stage markers has been limited because of procedural complexity and poor reproducibility. Some markers such as thrombomodulin (TM), thrombin‐antithrombin complex (TAT), plasmin‐α2‐antiplasmin complex (PAP), and t‐PA: PAI‐1 complexes (tPAI‐C) have been applied in the clinic. TM, one of the most commonly used indicators of endothelial injury, is located on vascular endothelium surfaces where it functions as an anticoagulant. TM has affinity for thrombin, forming a 1:1 thrombin‐thrombomodulin complex that inhibits fibrin formation, platelet activation, and protein S inactivation.^1^ Apart from its transmembrane form, TM also exists in soluble form in the plasma (sTM), which is probably produced from the cleaved transmembrane glycoprotein.^2^ In vitro studies demonstrate that sTM is released from endothelial cells following cell membrane injury caused by the action of neutrophil‐derived proteases and oxygen radicals.^3^ Elevated concentrations of sTM are observed in clinical conditions associated with vasculitis during their active phase.^4^, ^5^ In contrast to other popular endothelial markers, such as von Willebrand factor (vWF) or tissue‐type plasminogen activator (t‐PA), sTM does not have a circadian rhythm, and its expression does not increase with age, after exercise, or in acute response to a variety of biological stimuli. For this reason, plasma TM is likely a marker specific for endothelial lesion rather than endothelial activation.^1^, ^6^

Thrombin‐antithrombin complex is a molecular complex composed of thrombin and AT, a primary thrombin inhibitor, in a 1:1 ratio. Increased TAT indicates excess thrombin production and serve as a marker reflecting prothrombotic status. Increased thrombin levels signify activation of the blood coagulation cascade. However, thrombin levels cannot be measured directly due to extremely short half‐life in blood, whereas TAT has a half‐life of 3‐15 minutes, enabling direct measurement. Consequently, TAT could be used to indirectly assess thrombin generation as a molecular marker of activated coagulation and reflect ongoing condition of DIC.^7^, ^8^, ^9^, ^10^

Plasmin is a key enzyme of fibrinolysis, and activated blood coagulation results in increased formation of plasmin. Plasmin and α2‐antiplasmin form a stoichiometric 1:1 complex, which produces the plasmin‐α2‐antiplasmin complex (PAP) and neutralizes plasmin activity. The presence of PAP in plasma is therefore a direct indicator of the in vivo activity of plasmin and reflects a hyperfibrinolytic state.^8^ Lower PAP levels are associated with subsequent coronary events in patients with a history of MI.^11^ A previous study demonstrated that PAP is useful for predicting the risk of perioperative VTE and for identifying patients with a high risk of fatal perioperative PE.^12^ tPAI‐C is a complex of t‐PA with its type 1 inhibitor (PAI‐1) which has been reported to be of practical value in assessing risk of MI and VTE for both genders, especially for smokers or diabetes mellitus patients.^13^, ^14^ PAI‐1 is synthesized from the endothelial cells as an active molecule, but it then spontaneously converts to a latent form, which can be partially reactivated in vitro.^15^ Active form is secreted by cells and forms a stoichiometric 1:1 complex with the PA. Relative to the other serpins, active conformation of the PAI‐1 is the least stable.^15^ But most studies revealed that active PAI‐1 is a potential marker of VTE, in contrast to PAI‐1 antigen. But the measurement of active PAI‐1 is complex in vitro. Therefore, we choose the marker of tPAI‐C. In sum, TM, TAT, PAP, and tPAI‐C all have potential clinical usefulness for the diagnosis, monitoring, and clinical prognosis of thrombus. However, their clinical application is limited because ELISA, which is the classical detection method for these factors, is time‐consuming and has poor repeatability. The HISCL‐5000 chemiluminescence analyzer has the advantage of good repeatability, high sensitivity, high specificity, and simple operation. We have evaluated the clinical performance of this analyzer in this report.

## 2 MATERIALS AND METHOD

### 2.1 Samples

Commercially available normal and pathological lyophilized plasma samples were obtained from Sysmex Corporation. In addition, plasma samples referred to the laboratory for routine coagulation testing were also evaluated. This includes plasma from health examinations of patients with normal coagulation and biochemical (fasting serum glucose, lipids, liver and kidney functions, etc) profiles and patients with disseminated intravascular coagulation. Venous blood was collected into polymer evacuated tubes (BD, New York, USA), containing 0.109M sodium citrate (1vol./9vol.) according to international recommendations. Plasma was obtained by centrifugation at 2000 × *g* for 15 minutes and either tested within 2 hours or frozen and stored in aliquots at −20°C depending on the test requirements.

### 2.2 Analyzer

The HISCL‐5000 (Sysmex Corporation, Kobe, Japan) instrument is a fully automated analyzer. The TM, TAT, PAP, and tPAI‐C assays are one‐step or two‐step double‐antigen sandwich qualitative chemiluminescence enzyme immunoassays performed on a fully automated analyzer (HISCL‐5000; Sysmex Corporation, Kobe, Japan). Samples were tested according to the manufacturer's instructions with a total assay time of 17 minutes.

### 2.3 Assay

#### 2.3.1 Precision testing

System precision for TM, TAT, PAP, and tPAI‐C assays was based on the EP05‐A3 protocol of the CLSI.^16^ Intraassay variability was determined at three levels by measuring three patient samples (normal, deviant, and very deviant) 21 times. Interassay variability was evaluated by performing two tests on 10 separate days, with each test consisting of normal and pathological lyophilized plasma samples. For each of the studied parameters, intraassay variability and interassay variability were expressed as coefficients of variation (CV%), which were calculated as the standard deviation divided by the mean value.

#### 2.3.2 Carryover

For each test, two patient samples were selected: a sample with a low‐test result and a sample with a high‐test result. These samples were further divided into 3 “low” aliquots (L) and 3 “high” aliquots (H). Aliquots were loaded into the analyzer in the following order: H1, H2, H3, L1, L2, and L3. The carryover rate was calculated using the formula CR = (L1−L3)/(H3−L3) × 100%.

#### 2.3.3 Linearity analysis

Calibration curve linearity was determined using serial dilutions of a high‐concentration pool (H) and a low‐concentration pool (L). Six equally spaced concentration pools were prepared as follows: 5H, 4H + L, 3H + 2L, 2H + 3L, H + 4L, and 5L. Each dilution was analyzed in duplicate. The concentration of each pool was defined by the following formula, where the concentration of Pool L is *C*_L_, the volume of Pool L is *V*_L_, the concentration of Pool H is *C*_H_, and the volume of Pool H is *V*_H_: expectant concentration=(*C*_L_ × *V*_L_ + *C*_H_ × *V*_H_)/(*V*_L_ + *V*_H_). Outliers were identified according to the CLSI EP06‐A guidelines.^17^ Polynomial regression analysis was also performed.

## 3 REFERENCE RANGES

Normal reference ranges were verified by testing samples from 30 healthy individuals. If no more than three results (10%) fell outside the reference interval provided by the manufacturer, the interval was considered verified. Otherwise, normal reference ranges were established by testing samples from 200 healthy individuals.

## 4 STABILITY TESTING

Sample stability was evaluated by testing aliquots of patient samples placed for 0, 1, 3, 5, and 7 days at room temperature and in 4°C and −20°C environments.

## 5 INTERFERENCE STUDIES

Interference studies were performed to determine whether TM, TAT, PAP, and tPAI‐C measurements were affected by other substances such as hemoglobin (Interference Check A Plus, Sysmex Corporation, Kobe, Japan), triglycerides (Interference Check A Plus, Sysmex Corporation, Kobe, Japan), or bilirubin (free and conjugated forms; Interference Check A Plus, Sysmex Corporation, Kobe, Japan). Pooled plasma samples with normal and abnormal levels were mixed with hemoglobin, triglyceride, free bilirubin, and conjugated bilirubin to assess potential interference. The final concentration of hemoglobin used in these assays was 0, 51, 102,153, 204, 306, 357, 408, 459, and 510 mg/dL; the final concentration of triglycerides was 0, 149, 298, 447, 596, 745, 894, 1043, 1192, 1341, and 1490 FTU; and the final concentration of free and conjugated bilirubin was 0, 2.1, 4.2, 6.3, 8.4, 10.6, 12.7, 14.8, 16.9, 19.2, and 21.1 mg/dL.

### 5.1 Statistical analysis

The results are expressed as the mean value with SD when the data were normally distributed. Otherwise, the results are expressed as the median value with range. Statistical analysis was performed using SPSS 19.0 software (IBM, New York, USA).

## 6 RESULTS

### 6.1 Precision

Intraassay variability was determined, for each test by evaluating three levels of patient samples (normal, deviant, and very deviant) 21 times in the same day. As shown in Table 1, the coefficients of variation (CV%), calculated as standard deviations divided by mean values, were below 4.5% for all tested parameters in both the normal and abnormal ranges.

**Table 1:** Intraasay variability of the HISCL‐5000 Analyzer for TM, TAT, PAP, and tPAI‐C

	Level 1 (mean ± s)	CV(%)	Level 2 (mean ± s)	CV(%)	Level 3 (mean ± s)	CV(%)
TM	9.2 ± 0.15	1.60	10.4 ± 0.37	3.58	26.6 ± 0.38	1.43
TAT	1.1 ± 0.05	4.31	15.2 ± 0.17	1.14	89.7 ± 1.38	1.54
PAP	1.823 ± 0.04	2.26	6.582 ± 0.10	1.56	10.560 ± 0.20	1.91
tPAI‐C	7.8 ± 0.11	1.47%	17.2 ± 0.33	1.92	22.4 ± 0.28	1.26

Abbreviations: PAP, plasmin‐α2‐antiplasmin; TAT, thrombin‐antithrombin; TM, thrombomodulin; tPAI‐C, t‐PA: PAI‐1 complex.

Interassay variability was evaluated by performing two tests on 10 separate days, with each test consisting of two replicates of normal and pathological lyophilized plasma samples. As shown in Table 2, the CV% was below 2.0% for all tested parameters.

**Table 2:** Interassay variability of the HISCL‐5000 Analyzer for TM, TAT, PAP, and tPAI‐C

	Level 1 (mean ± s)	CV(%)	Level 2 (mean ± s)	CV(%)
TM	9.2 ± 0.15	1.60	10.4 ± 0.37	3.58
TAT	1.1 ± 0.05	4.31	15.2 ± 0.17	1.14
PAP	1.823 ± 0.04	2.26	6.582 ± 0.10	1.56
tPAI‐C	7.8 ± 0.11	1.47%	17.2 ± 0.33	1.92

Abbreviations: PAP, plasmin‐α2‐antiplasmin; TAT, thrombin‐antithrombin; TM, thrombomodulin; tPAI‐C, t‐PA: PAI‐1 complex.

### 6.2 Carryover

Sample carryover was investigated by measuring 3 aliquots of low‐level samples and 3 aliquots of high‐level samples. As shown in Table 3, the carryover rate was below 1% for all tested parameters.

**Table 3:** Carryover rate of the HISCL‐5000 Analyzer for TM, TAT, PAP, and tPAI‐C

	TM	TAT	PAP	tPAI**‐**C
H1	32.1	17	2.094	31
H2	31.3	16.6	2.038	30.8
H3	32.1	16.9	2.046	31.6
L1	11.4	2	0.182	4.2
L2	11.3	2	0.177	4.2
L3	11.3	1.9	0.181	4.2
Carryover rate（%）	0.48	0.67	0.05	0.00

Abbreviations: PAP, plasmin‐α2‐antiplasmin; TAT, thrombin‐antithrombin; TM, thrombomodulin; tPAI‐C, t‐PA: PAI‐1 complex.

### 6.3 Linearity analysis

**Figure 1:**
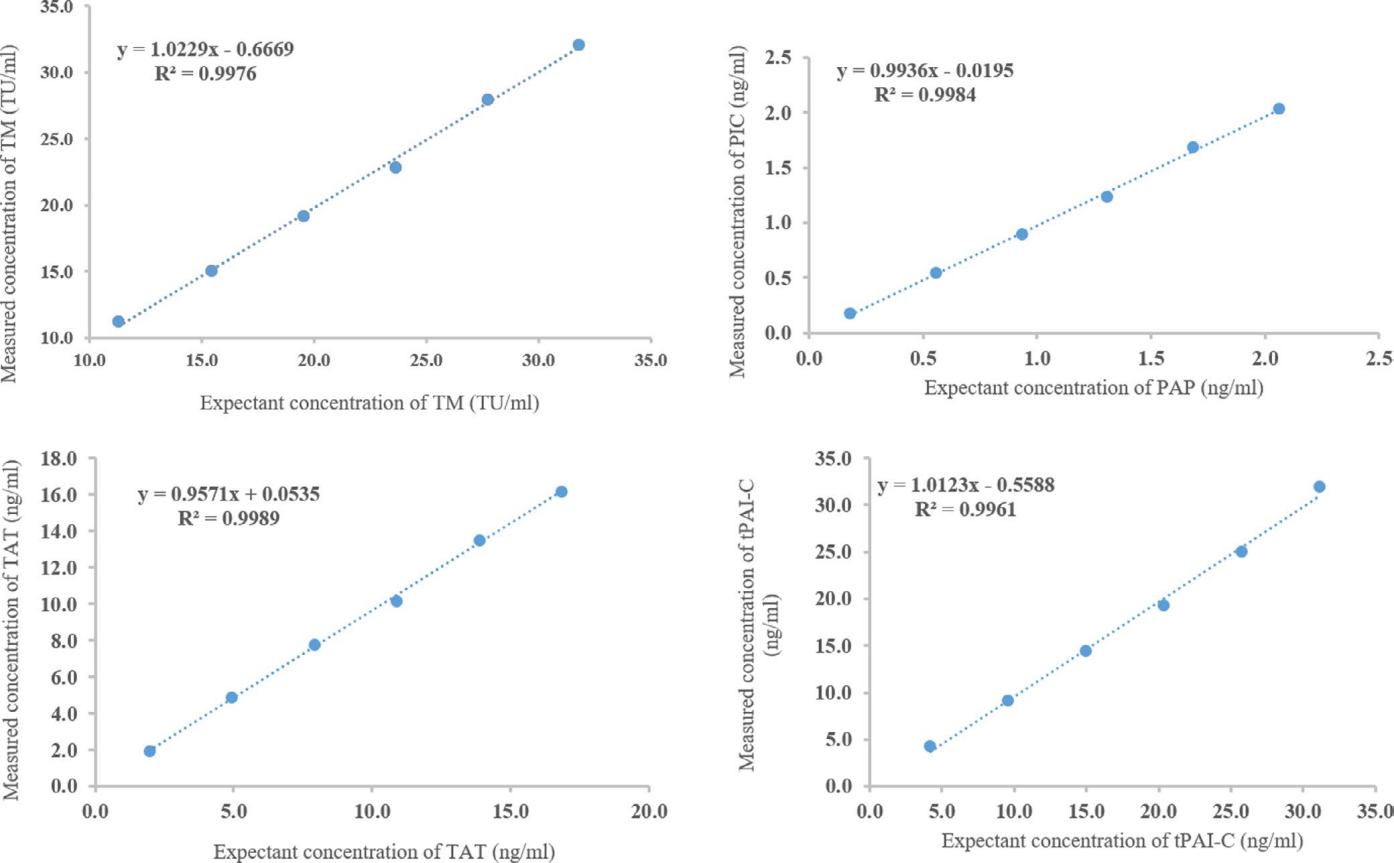
Calibration curve linearity for thrombomodulin, thrombin‐antithrombin, plasmin‐α2‐antiplasmin, and t‐PA: PAI‐1 complex

The best fitting polynomial regression model was the first‐order model: *Y = a* + *bX*. As shown in Figure 1, the correlation indexes were between 0.998 and 0.999.

### 6.4 Reference ranges

Normal reference ranges were verified for all tested parameters by investigating plasma samples from 30 apparently healthy individuals. As shown in Table 4, reference intervals provided by the manufacturer were verified successfully for all parameters except for tPAI‐C, for which more than three results (10%) fell outside the provided reference intervals provided by the manufacturer. Therefore, the normal reference range of tPAI‐C was established by testing samples from 200 healthy individuals (100 males and 100 females). This established reference interval for tPAI‐C is shown in Table 5.

**Table 4:** Normal reference range verification for TM, TAT, PAP, and tPAI‐C

	Reference ranges	Sample size	Fell outside size
TM	3.8‐13.3 TU/mL	30	0
TAT	<4.0 ng/mL	30	2
PAP	<0.8 ng/mL	30	3
tPAI‐C (Male)	6.2‐14 ng/mL	30	18
tPAI‐C (Female)	3.7‐9.3 ng/mL	30	6

Abbreviations: PAP, plasmin‐α2‐antiplasmin; TAT, thrombin‐antithrombin; TM, thrombomodulin; tPAI‐C, t‐PA: PAI‐1 complex.

**Table 5:** Normal reference ranges of tPAI‐C

	Reference ranges	Sample size
tPAI‐C (Male)	1.07‐11.17 ng/mL	100
tPAI‐C (Female)	1.18‐8.65 ng/mL	100

Abbreviation: tPAI‐C, t‐PA: PAI‐1 complex.

### 6.5 Patients’ sample stability

**Figure 2:**
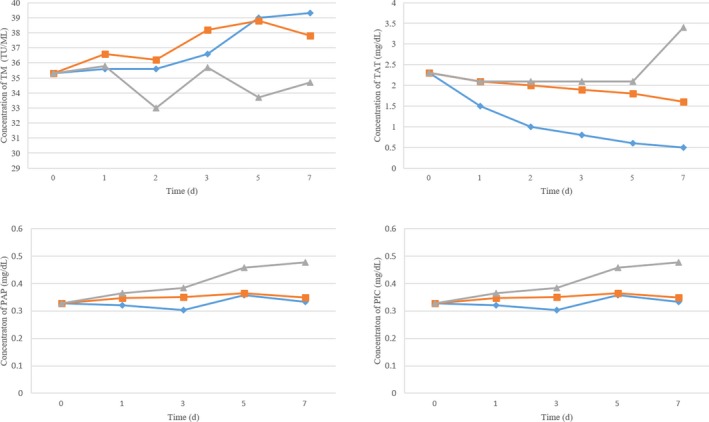
Sample stability for thrombomodulin, thrombin‐antithrombin, plasmin‐α2‐antiplasmin, and t‐PA: PAI‐1 complex

Patients’ sample stability was evaluated by testing aliquots of patient samples placed for 0, 1, 3, 5, and 7 days at room temperature and in 4°C and −20°C environments. As shown in Figure 2, TM is stable for 2 days at room temperature but unstable at the other tested temperatures. TAT is stable for 5 days at 4°C and −20°C but has poor stability at room temperature. PAP and tPAI‐C are both stable for 3 days at all three temperatures investigated.

### 6.6 Interference studies

**Figure 3:**
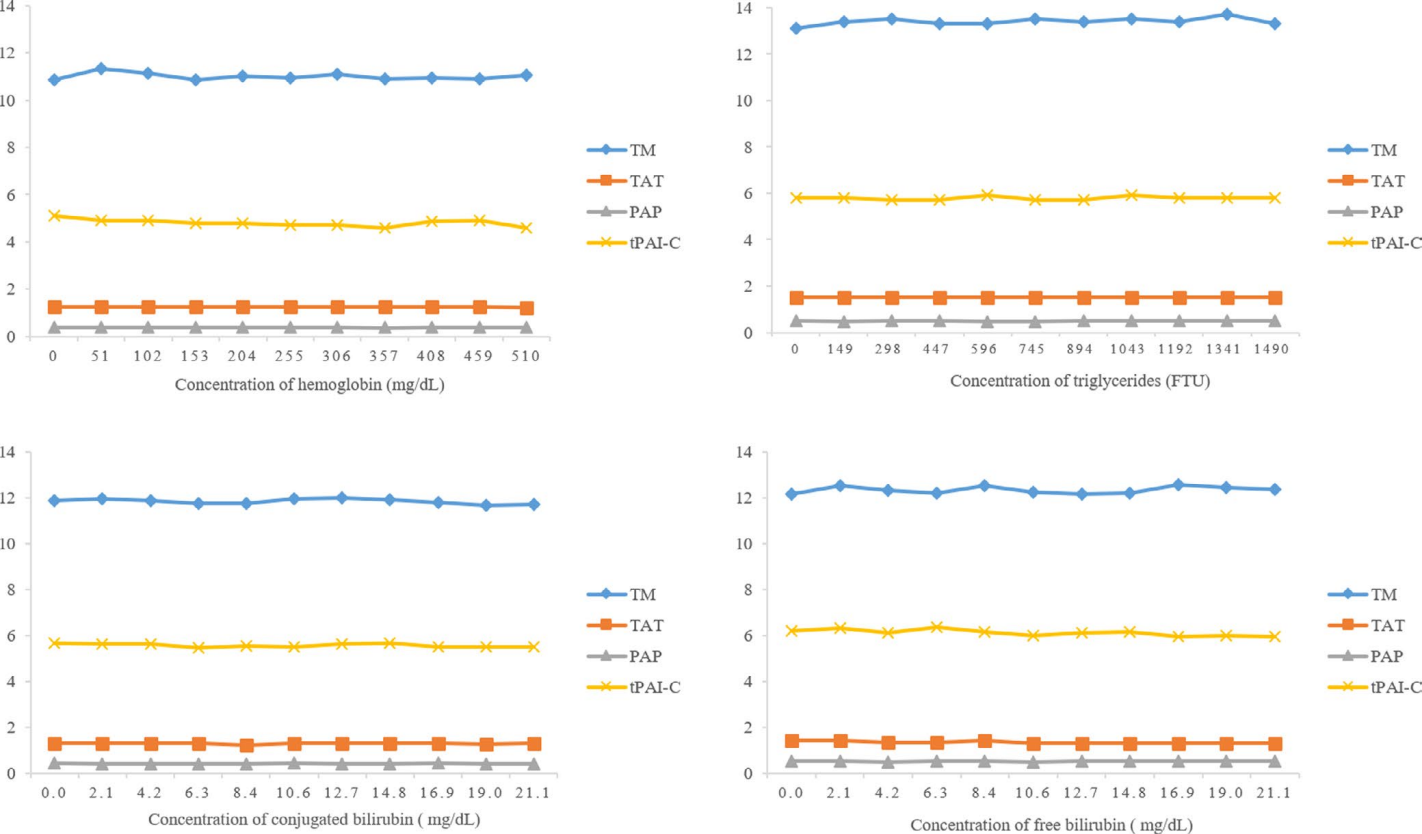
Interference studies of hemoglobin, triglycerides, and bilirubin on low level of thrombomodulin, thrombin‐antithrombin, plasmin‐α2‐antiplasmin, and t‐PA: PAI‐1 complex

Interference studies were performed to evaluate the effects of hemoglobin, triglycerides, and bilirubin on tested parameters. As shown in Figure 3, the presence of hemoglobin, triglycerides, free bilirubin, and conjugated bilirubin does not affect the reliability of any tested parameters.

## 7 DISCUSSION

The classical detection method for TM, TAT, PAP, and tPAI‐C is enzyme‐linked immunosorbent assay (ELISA), which is gold standard method. But ELISA is time‐consuming and has poor repeatability. With the development of high‐sensitivity chemiluminescence immunoassay, we can test these factors automatically. Therefore, we performed analytical verification of TM, TAT, PAP, and tPAI‐C using a high‐sensitivity chemiluminescence analyzer according to the specifications of the Clinical and Laboratory Standards Institute (CLSI).

The reference intervals provided by the manufacturer were verified successfully for all parameters except for tPAI‐C, for which reference intervals should be established according to gender.

Sample stability assessment revealed that TM is stable for 2 days at room temperature but lacks stability at colder temperatures, whereas TAT is stable for 5 days in 4°C and −20°C but has poor stability at room temperature. PAP and tPAI‐C are both stable for 3 days at all three temperatures. Therefore, samples should be analyzed after centrifugation as soon as possible, and frozen preservation is not recommended.

In conclusion, verification data for precision, carryover, linearity, and interference revealed that the performance of the analyzer tested here is acceptable. However, the reference interval of tPAI‐C should be established by each laboratory, and a comparison using another method should be performed in the future.

## CONFLICT OF INTEREST

The authors have no conflicts of interests.
